# Antibacterial Capability, Physicochemical Properties, and Biocompatibility of nTiO_2_ Incorporated Polymeric Scaffolds

**DOI:** 10.3390/polym10030328

**Published:** 2018-03-16

**Authors:** Cijun Shuai, Chenying Shuai, Pei Feng, Chengde Gao, Shuping Peng, Youwen Yang

**Affiliations:** 1 Jiangxi University of Science and Technology, Ganzhou 341000, China; shuai@csu.edu.cn; 2 State Key Laboratory of High Performance Complex Manufacturing, College of Mechanical and Electrical Engineering, Central South University, Changsha 410083, China; shuaichenying@csu.edu.cn (C.S.); fengpei@csu.edu.cn (P.F.); gaochengde@csu.edu.cn (C.G.); 3 Key Laboratory of Organ Injury, Aging and Regenerative Medicine of Hunan Province, Changsha 410008, China; 4 The Key Laboratory of Carcinogenesis of the Chinese Ministry of Health, Xiangya Hospital, Cancer Research Institute, Central South University, Changsha 410008, China

**Keywords:** nano titanium dioxide, antibacterial capability, cellular attachment, polymeric scaffolds

## Abstract

Postoperative infection is a common risk which brings about failure in bone transplantation. In this study, nano titanium dioxide (nTiO_2_) was incorporated into Polyetheretherketone/polyglycolicacid (PEEK/PGA) blends to construct antibacterial scaffolds via selective laser sintering. Antibacterial capability was assessed using *Escherichia coli* (*E. coli*) and *Staphylococcus aureus* (*S. aureus*). The results demonstrated that the scaffolds with nTiO_2_ presented an effective antibacterial activity, which might be attributed to that nTiO_2_ would do the mechanical and oxidative damage to bacteria by occurring contact actions and generating reactive oxygen species (ROS), and thus killed bacteria from structure and function. Moreover, nTiO_2_ could enhance the tensile strength and modulus of scaffolds due to the reinforcing effect and its uniform disperse. And the cell culture experiments showed that nTiO_2_ stimulated cellular attachment and proliferation. Besides, it also elevated the hydrophily and thermal stability of scaffolds. These results suggested that the polymeric scaffolds incorporated nTiO_2_ had potential application in bone tissue engineering.

## 1. Introduction

There exists a risk of bacterial infections after artificial bone transplantation [1,2]. It is a common method to use antibiotics for preventing bacterial infections [3,4]. However, their long-term application would bring about toxic and adverse effects including hypersensitivity, immune suppression, and allergic reaction [5,6,7]. In addition, the abuse of antibiotics would cause drug resistance in bacteria [8]. Therefore, it is necessary to develop antibacterial scaffolds to deal with the implant-associated bacterial infections. A potential strategy is to incorporate antibacterial materials into scaffolds [9,10,11,12].

Acknowledged as a typical inorganic antibacterial biomaterial, nano titanium dioxide (nTiO_2_) has been given much attention due to its strong and broad-spectrum antimicrobial action [13,14,15,16]. Moreover, nTiO_2_ presents good biocompatibility, excellent biosecurity, and extraordinary hydrophilicity [17,18,19]. Besides, it is a desired candidate as a reinforcing filler in a polymeric matrix [20]. Further, the strength of bone-titanium integrations is considerably greater compared with other nanoparticles [21]. Polyetheretherketone/polyglycolicacid (PEEK/PGA) blends have potentially used in bone repair because of their remarkable processability, biocompatibility, and degradation property [22]. As an additive manufacturing method, selected laser sintering (SLS) is able to construct scaffolds with complex shapes and accurate interconnected porous structures [23,24,25].

Recently, Wu et al. fabricated TiO_2_/Poly(lactic-co-glycolic acid) composite biomaterial by a sol–gel method and confirmed that the composites possessed biocompatibility and antibacterial properties [26]. González-García et al. prepared Polyurethanes/TiO_2_ hybrid via a sol–gel reaction and found that the hybrids exhibited improved thermal and mechanical properties compared with the polyurethanes matrix [27]. Roether et al. developed Poly(glycerol sebacate)/Poly(3-hydroxybutyrate)-TiO_2_ nanocomposite through solvent casting and discovered that adding TiO_2_ into the scaffolds led to an encouraging improvement in mechanical properties and wettability [28]. However, scarce works focused on the comprehensive performances of nTiO_2_ contained scaffolds fabricated by selective laser sintering.

In this study, nTiO_2_ was incorporated into PEEK/PGA blends. The scaffolds with nTiO_2_ were prepared via selective laser sintering. The effect of nTiO_2_ on antibacterial activity against *Escherichia coli* (*E. coli*) and *Staphylococcus aureus* (*S. aureus*) was evaluated. And the microstructures and thermal properties of the scaffolds were characterized. Meanwhile, their mechanical properties, degradation behavior, and cell response were also investigated.

## 2. Materials and Methods

### 2.1. Materials and Scaffolds Preparation

PEEK (powder size 20–50 μm, Mw: 270,000 g/mol, Purity ≥99.5%) was supplied by Dongguan Guanhui Plastic Materials Co., Ltd. (Dongguan, China). PGA (powder size ~40 μm, Mw: 1,000,000 g/mol, Purity ≥99.5%) was purchased from Shenzhen Polymtek Biomaterial Co., Ltd. (Shenzhen, China). nTiO_2_ (rutile) (powder size ~30 nm, Purity ≥99.9%) was obtained from Nanjing Emperor Nano Material Co., Ltd. (Nanjing, China). And all the other agents were analytical reagents.

**Figure 1:**
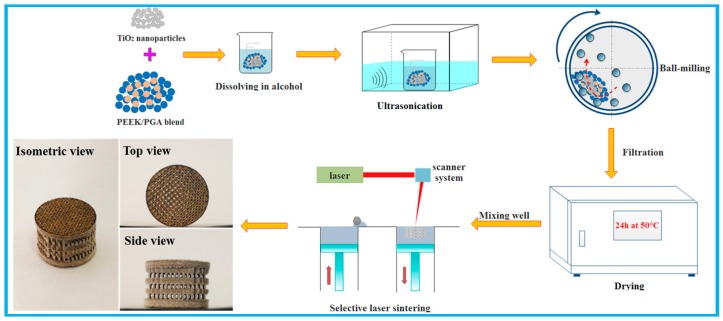
Schematic of the scaffold preparation process.

The overall preparation procedure of the scaffolds was represented in Figure 1. Firstly, TiO_2_ nanoparticles and PEEK/PGA blended powders (8:2 mass ratios) were dissolved in alcohol and dispersed by bath ultrasonication over 20 min, respectively. Subsequently, nTiO_2_ solution was added into PEEK/PGA solution, and then they were homogenized via ultrasonic cleaner for 30 min. To further improve the dispersion of nTiO_2_, the mixed solution was grinded using a variable frequency planet-type grinding mill for 60 min. After that, the obtained solution was filtered through qualitative filter paper and was exsiccated at 50 °C for 24 h in a drying cabinet. Finally, a series of PEEK/PGA-nTiO_2_ mixed powders were obtained for scaffold preparation, with nTiO_2_ contents of 0, 1, 3, 5, and 7 wt %, respectively.

The scaffolds with nTiO_2_ loading of 0 wt % (PEEK/PGA), 1 wt % (PEEK/PGA-1%nTiO_2_), 3 wt % (PEEK/PGA-3%nTiO_2_), 5 wt % (PEEK/PGA-5%nTiO_2_), and 7 wt % (PEEK/PGA-7%nTiO_2_) were fabricated via selective laser sintering. The system consisted of a CO_2_ laser, scanner system, sintering platform and corresponding control system. During sintering, the laser beam selectively scanned the powders according to the cross-sectional area of scaffolds. The sintering platform was subsequently moved down a layer thickness, and the sintering procedure was repeated. The interconnected porous scaffold with an overall size of 13 mm × 10 mm (diameter × height) was manufactured, as presented in Figure 1.

### 2.2. Characterization

The phase constituents of the scaffolds were determined using an X-ray diffractometer (XRD, Rigaku Co., Tokyo, Japan). The XRD data were recorded at the scanning rate of 8°/min in 2*θ* range from 10° to 80°. Fourier transform infrared spectroscopy (FTIR) analysis was performed with Nicolet 6700 spectrometer (Thermo Scientific Co., Madison, WI, USA) in the wavenumber range from 4000 cm^−1^ to 600 cm^−1^. Moreover, the scaffolds were analyzed through a scanning electron microscopy (SEM; JEOL JSM 7600F, Tokyo, Japan). All the specimens were sputtered by platinum (JFC-1600, Jeol Co., Tokyo, Japan). And the elemental constituents were investigated by an energy dispersive spectroscopy (EDS).

Tensile strength of the scaffolds was tested with an electronic universal testing machine at a crosshead speed of 0.5 mm/min (WD-D1, Shanghai Zhuoji Instruments Co., Ltd., Shanghai, China). The scaffold samples (10 × 10 × 5 mm^3^) were tested at ambient temperature and five samples for each group were tested. Then the mean value was calculated. Besides, the tensile modulus was obtained through the initial slope of the stress-strain curve.

The thermal stability of the scaffolds was investigated by thermogravimetric analysis (TGA) with STA-200 Instruments (Optoma Europe Ltd., Hertfordshire, UK). Specimens (approximately 15 mg) were sealed in aluminum pan. Then temperature was scanned from 25 °C to 700 °C at a heating rate of 10 °C/min under an inert atmosphere. Dynamic differential scanning calorimetry (DSC) experiments were conducted under a nitrogen atmosphere. Samples (approximately 10 mg) were heated from 25 °C to 700 °C. The steps were implied at a constant rate of 10 °C/min, and transition temperatures were regarded as the peak minimum of calorimetric curve.

Water contact angle of scaffolds was measured to evaluate the wetting property using a KSV CAM 200 optical contact angle meter (KSV Instruments Ltd., Helsinki, Finland). Deionized water drops, which were used as probe liquid, were place on the samples surface at room condition. Also, five independent measurements for each group were averaged to calculate contact angle.

### 2.3. Antibacterial Activity Assay

The antibacterial capability of scaffolds was assessed by plate count method against *E. coli* (ATCC, 25922) and *S. aureus* (ATCC, 12600). Prior to the experiments, all the scaffolds were sterilized in a steam autoclave and desiccated on superclean bench. The 10 µL solution with *E. coli* and 10 µL solution with *S. aureus* at a density of 1 × 10^7^ CFU/mL was added into each scaffold, followed by using sterilized polyethylene films to cover their surface. After anaerobic cultivation for 24 h at 37 °C, the bacteria on scaffolds were collected to new agar plates, and then the number of viable bacteria was counted using plate count method. The antibacterial rate was estimated following the equation: *A*% = [(*B* − *E*)/*B*] × 100%, where *A*% was the antibacterial rate, *E* and *B* were the bacterial number of the experimental groups and blank control group, respectively. Additionally, the scaffolds without TiO_2_ were used as blank control. Five tests were conducted to obtain the average value.

### 2.4. Cell Culture

Human osteoblast-like cells (MG-63) were used to investigate the compatibility of the scaffolds. The cell lines were maintained and cultured in the Dulbecco’s modified Eagle’s medium supplemented with 10% FBS and 1% penicillin/streptomycin. The scaffolds were placed in a 12-well plate after being sterilized in the autoclave. After that, the scaffolds seeded with 2 × 10^4^ cell/cm^2^ of cells were cultivated in a humid incubator with 5% CO_2_ atmosphere at 37 °C, and culture medium was refreshed every other day. After predetermined incubation time, the scaffolds were fetched out, fixed in 4% glutaraldehyde for 1 h, and then dehydrated using gradient ethanol. In the end, cell attachment and spread were investigated under SEM after they were entirely dried.

At 1, 3, and 5 days of incubation, the scaffolds were rinsed with phosphate buffer solution (PBS) twice after being removed out, and then adherent cells were immobilized with paraformaldehyde, permeabilized with 0.5% Tween 20. Afterwards, the cells were purged again and incubated into PBS including 4 µM EthD-1 and 2 µM calcein AM for 25 min. In the end, the cells were observed under a fluorescence microscope.

In addition, microculture tetrazolium test (MTT) assay was performed to estimate the cell proliferation. After being fostered for 1, 3, and 5 days, 20 μL of MTT solution was dropped into cell culture plates. Subsequently, it was kept at 37 °C for 3 h, and formazan crystals were completely dissolved with 200 mL dimethyl sulphoxide (DMSO) after throwing off the supernatants. Finally, the absorbency was measured with a microplate reader at 570 nm. Five parallel experiments for each group were numerated. Moreover, the culture medium was used as blank group.

### 2.5. In Vitro Degradation Study

The in-vitro degradation behavior of the scaffolds was evaluated using PBS solution (pH = 7.4). All the specimens were first weighed (*M_i_*). Whereafter, they were immersed in the solution and incubated at 37 °C for prearranged periods (7, 14, 21, and 24 days). The PBS was renewed every 3 days. At each prescribed time, the specimens were withdrawn and mildly rinsed with deionised water, and then entirely dried at 40 °C to obtain final weight (*M_f_*). The weight loss (*W_l_*) were determined using following formula:

*W_l_* = (*M_i_* − *M_f_*)/*M_i_* (1)

### 2.6. Statistical Analysis

All experimental data were listed as the mean ± standard deviation (SD). One way ANOVA was implemented to determine statistical significance. In all analyses, *p* < 0.05 was considered to be statistically significant.

## 3. Results and Discussion

### 3.1. Microstructural Characteristics

**Figure 2:**
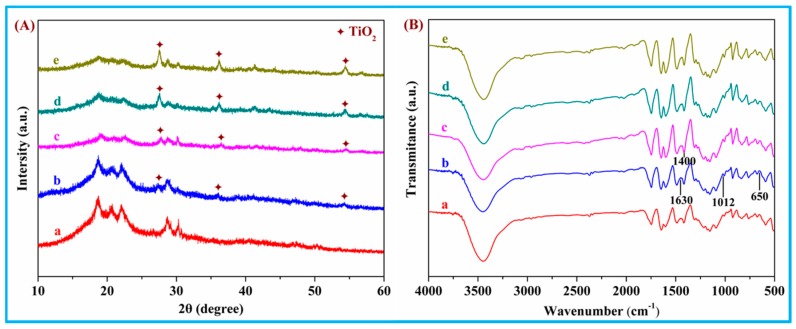
(**A**) X-ray diffractometer (XRD) spectra and (**B**) Fourier transform infrared spectroscopy (FTIR) spectra for the scaffolds with (a) 0 wt %; (b) 1 wt %; (c) 3 wt %; (d) 5 wt %; and (e) 7 wt % nTiO_2_.

The XRD pattern of PEEK/PGA-nTiO_2_ scaffolds loading different nTiO_2_ concentration was shown in Figure 2A. PEEK phase and PGA phase, as the predominant components of the scaffolds, were distinctly displayed in all patterns. XRD diffraction peaks around 2*θ* of 27.42°, 36.10°, and 54.2° could be indexed to the characteristic peaks (101), (004), and (200) of rutile TiO_2_. Meanwhile, with the increase of nTiO_2_, these peaks increased in intensity. In addition, no extra peaks of other phase were visible. In fact, a rise in crystallinity was found for scaffolds with low nTiO_2_ contents, whereas the reverse trend was observed at high nTiO_2_ contents. The results hinted a change from promotion to retardation in the crystallization rate of the polymer with increasing nanoparticle concentration. Thus, the nucleating effect of the nanoparticles likely led to higher degree of crystallinity at low nTiO_2_ concentration, whereas the increased nTiO_2_-matrix interactions could prevail over the nucleation effect at higher nanofiller concentration. An analogous phenomenon has been reported for PEEK composites reinforced with inorganic WS_2_ nanoparticles, where the nucleation of the polymer crystals was favored at low nanofiller contents [29].

FTIR spectra of PEEK/PGA-nTiO_2_ scaffolds were recorded (Figure 2B). The spectrum of nTiO_2_ displayed peaks at 1630 and 1012 cm^−1^ arising from the bending vibration of coordinated H_2_O and Ti-OH. The peak at ~650 cm^−1^ was related to the Ti-O-Ti stretching motion, and that at ~1400 cm^−1^ corresponded to nTiO_2_ lattice vibrations. The results demonstrated that there was a successful combination of nTiO_2_ in the scaffolds.

**Figure 3:**
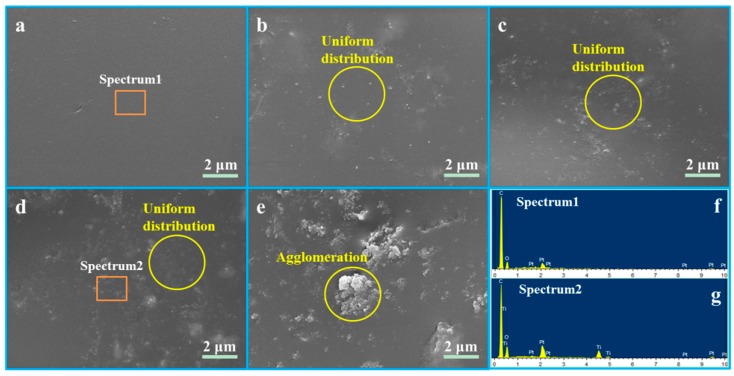
Morphology of (**a**) PEEK/PGA scaffold, (**b**) PEEK/PGA-1%nTiO_2_ scaffold, (**c**) PEEK/PGA-3%nTiO_2_ scaffold, (**d**) PEEK/PGA-5%nTiO_2_ scaffold, (**e**) PEEK/PGA-7%nTiO_2_ scaffold, and (**f**), (**g**) energy dispersive spectroscopy (EDS) spectra of the marked region.

The sintered surface of the scaffolds was characterized by SEM, and the composition of the PEEK/PGA and PEEK/PGA-5%nTiO_2_ scaffolds was detected by EDS with orange square area (Figure 3). The morphology of PEEK/PGA scaffolds appeared glossy and flat. After the incorporation of nTiO_2_, some particles arose in matrix. Moreover, the particles dispersed uniformly in the polymeric matrix when filler concentration was no more than 5 wt %, and they were evaluated using EDS (Figure 3g). The emergence peaks of Ti proved that these particles were nTiO_2_. Nevertheless, the nTiO_2_ formed agglomeration when further increasing its concentration (Figure 3e). The results might be due to that excessive nTiO_2_ could reduce the distance between nanoparticles, and thus increased their interaction force and the chance of contacts [30,31].

### 3.2. Thermal Stability and Hydrophilicity

**Figure 4:**
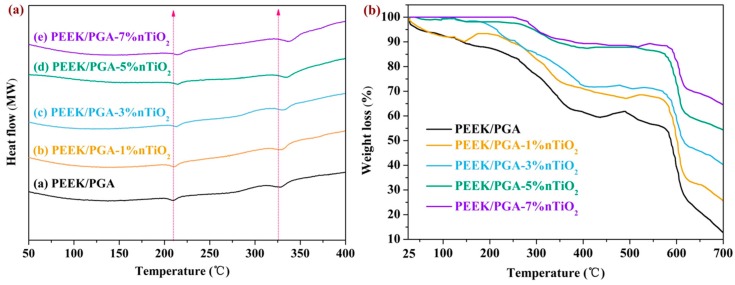
(**a**) Differential scanning calorimetry (DSC) curve and (**b**) thermogravimetric analysis (TGA) curve of the scaffolds with different nTiO_2_ contents.

DSC was utilized to assess the effects of nTiO_2_ on melting behavior of scaffolds, with results depicted in Figure 4a. DSC curves of PEEK/PGA scaffolds exhibited two apparent endothermic peaks at approximately 218 °C and 337 °C, which accorded with endothermic peaks of PGA and PEEK [32,33]. Moreover, the melt temperatures of scaffolds were increased with increasing nTiO_2_ compared with those of the scaffolds without nTiO_2_. The results were mainly because of the nucleation effect of TiO_2_ nanoparticles, since they could hasten the development of nucleus [34].

Thermal characterization of the scaffolds was investigated using TGA analysis (Figure 4b). It appeared that the scaffolds with nTiO_2_ and the scaffolds without nTiO_2_ decomposed by a two-step process, and the TGA profile of the scaffolds with nTiO_2_ scaffolds shifted to high temperature compared with that of the scaffolds without nTiO_2_. Moreover, the scaffolds with nTiO_2_ showed less weight loss than the scaffolds without nTiO_2_. The results suggested that the scaffolds with nTiO_2_ could elevate thermal stability. It was believed that nTiO_2_ could act as a barrier against the transportation of decomposed product [35]. On the other hand, nTiO_2_ possessed a high thermal conductivity, which made contributions to the heat dissipations within the polymeric matrix [36].

**Figure 5:**
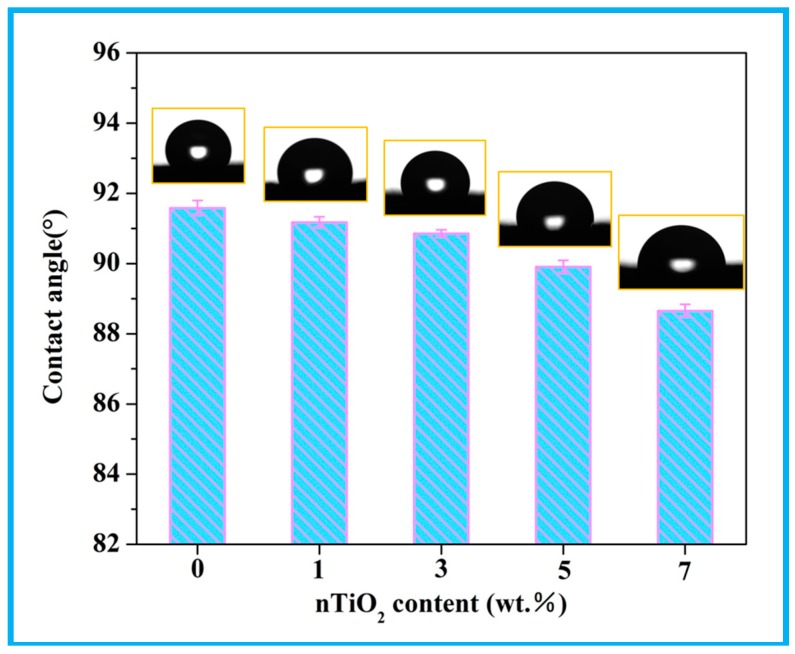
Water contact angle of the scaffolds with different nTiO_2_ contents.

The hydrophilicity of scaffolds is well-known as a crucial factor to determine cell response, which could be assessed by water contact angle (Figure 5). It could be visible that the contact angle reduced with increasing nTiO_2_, hinting that nTiO_2_ would improve the hydrophilicity of scaffolds. The cause might be that nTiO_2_ was hydrophilic owing to the existence of unsaturated reactive hydroxyl groups (-OH) [37].

### 3.3. Antibacterial Activity

**Figure 6:**
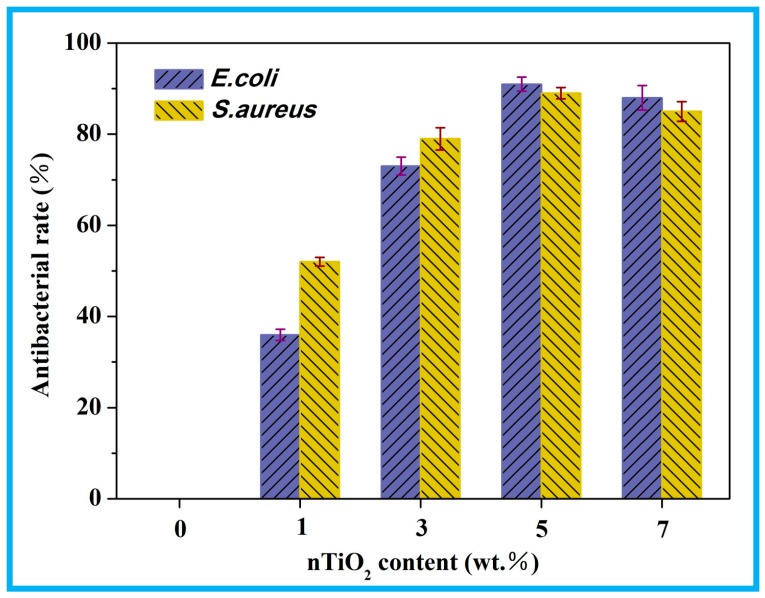
Antibacterial rate of the scaffolds with different nTiO_2_ contents.

The antibacterial capability of the scaffolds was explored against *E. coli* and *S. aureus* (Figure 6). As could be observed, the scaffolds without nTiO_2_ did not exhibit antimicrobial activity. While the scaffolds with nTiO_2_ presented antibacterial activity, and their antibacterial activity rose upon increasing nTiO_2_, displaying a maximum at 5 wt % nTiO_2_, and then declined. The results implied that nTiO_2_ played a vital role in antibacterial capability and could be explained that the overall interaction between nanofiller and bacteria would be weak at low nTiO_2_ contents [38]. While at high contents nanoparticle clusters reduced the effective surface-to-volume ratio of nanofiller, thus the overall interaction between nTiO_2_ and bacteria was also decreased [39].

**Figure 7:**
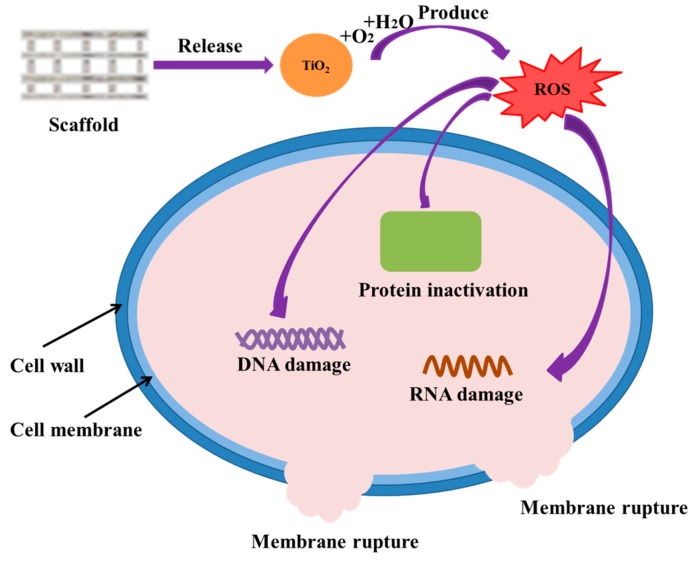
Possible antibacterial mechanisms of PEEK/PGA-nTiO_2_ scaffolds.

The possible antibacterial mechanisms were that (Figure 7): nTiO_2_ could react with water and oxygen, thus generating active oxygen species (ROS). ROS would make bacteria produce oxidative stress when its concentrations exceed the scavenging activity of bacterial antioxidant defense system, subsequently damaging the structure and function of the bacteria [40]. Besides, the contact action between nTiO_2_ and bacterial cell wall would generate mechanical stress, leading to the deformation of bacterial cell membrane [41]. Then nTiO_2_ would be absorbed into the bacteria and damage them from the interior.

### 3.4. Mechanical Properties

**Figure 8:**
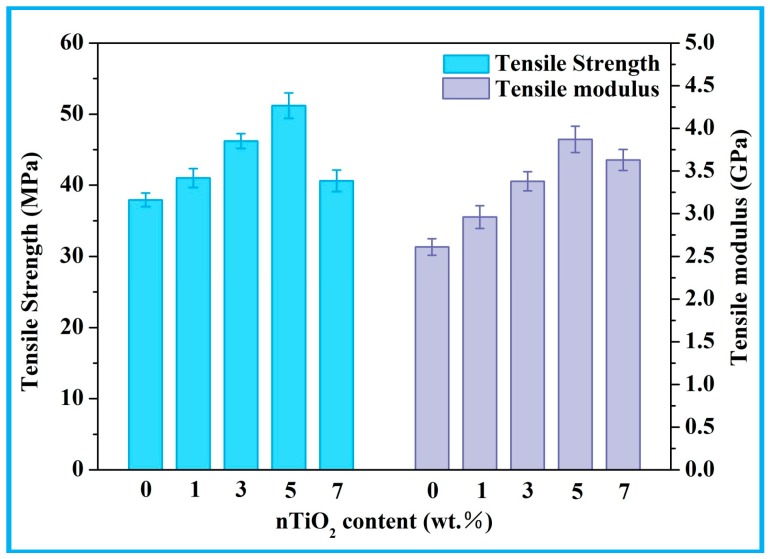
Tensile strength and modulus of the scaffolds with different nTiO_2_ contents.

The mechanical properties of PEEK/PGA-nTiO_2_ scaffolds were examined (Figure 8). Their tensile strength and modulus kept increasing when nTiO_2_ content was until 5 wt %. The incorporation of 5 wt % nTiO_2_ enhanced tensile strength to 51.20 MPa, being 35% higher than that of PEEK/PGA scaffolds. At the same time, tensile modulus increased to 3.87 GPa, which was 48% higher than that of polymeric scaffold. The improvements were owing to the reinforcement effect and uniformly-dispersed nTiO_2_. Noted that tensile strength and modulus would increase with inorganic filler contents were common in polymeric matrix [42,43,44]. However, when nTiO_2_ were excessive, the variation tendency of mechanical properties started to reverse. It could be explained that nTiO_2_ occurred agglomeration with its increase and leading to inhomogeneous dispersion. The results of the antibacterial experiments and mechanical experiments indicated that the optimal nTiO_2_ content was 5 wt % in this study. Therefore, the comprehensive performances of the scaffolds with 5 wt % nTiO_2_ were evaluated in follow-up experiments.

### 3.5. In Vitro Degradation

**Figure 9:**
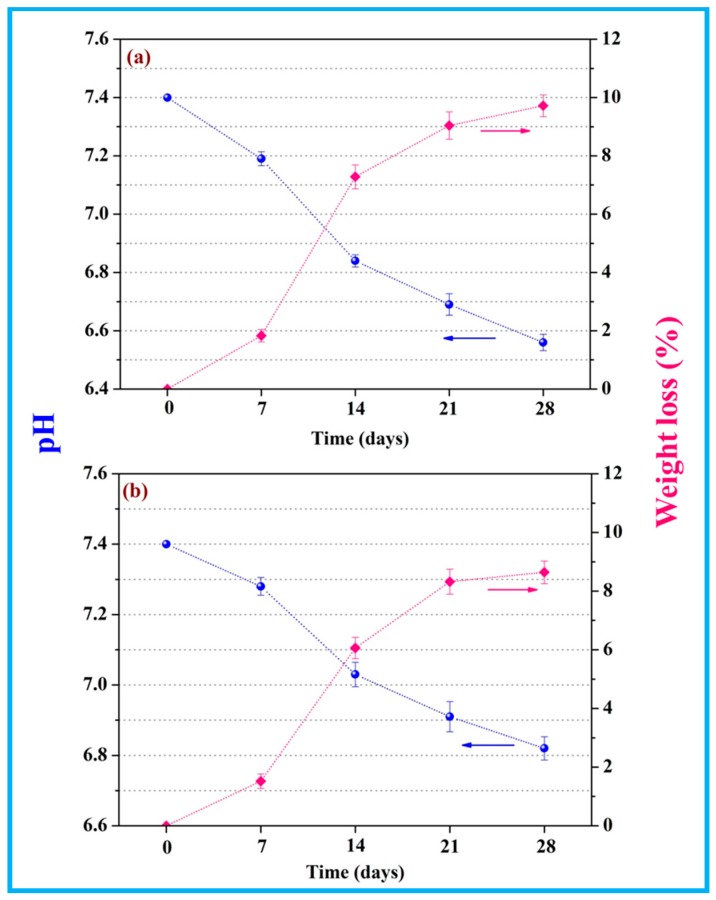
pH value and weight loss of (**a**) PEEK/PGA scaffolds and (**b**) PEEK/PGA-5%nTiO_2_.

The pH of scaffolds during in-vitro degradation was depicted in Figure 9. It decreased from 7.4 on account of the released acidic degradation product of PGA. In the meantime, it was evident that pH of the scaffolds with nTiO_2_ dropped slowly compared with that of the scaffolds without nTiO_2_. This was because the degradation of PGA was composed of a hydrolysis process, which would increase acid atmosphere, and hence its pH would present a downward trend. While the incorporation of nTiO_2_ provided a buffering effect that the scaffolds with nTiO_2_ could absorb water and then formed titanium hydroxide (Ti-OH groups) [45], consequently prevented the drop in pH.

In-vitro degradation was evaluated by weight loss. As the immersion time prolonged, weight loss of the scaffolds gradually increased. After degrading for 28 days, weight loss of the scaffolds with nTiO_2_ present a bit lower than that of the scaffold without nTiO_2_ (8.64% and 9.72%, respectively). It might be ascribed to that titanium hydroxide (Ti-OH groups) formed on the surface of nTiO_2_ would neutralize the acid degradation products of PGA [46], leading to the decrease of PGA degradation.

### 3.6. Biocompatibility Studies

**Figure 10:**
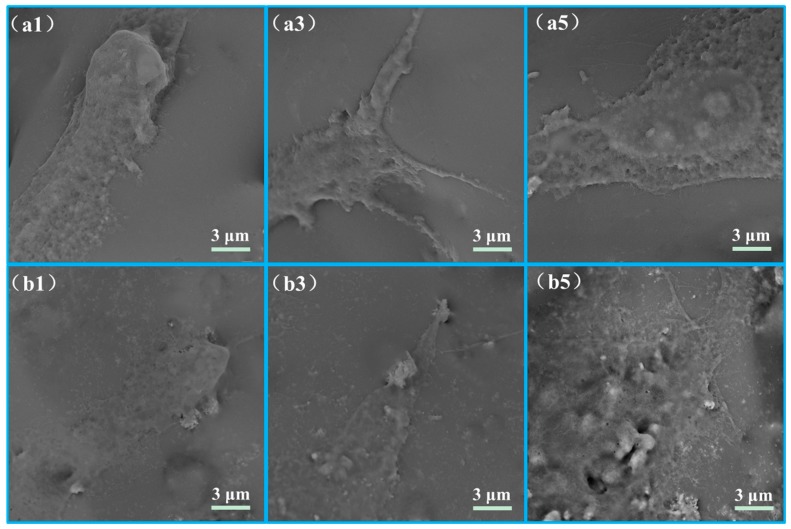
Cells on the (**a1**–**a5**) PEEK/PGA and (**b1**–**b5**) PEEK/PGA-5%nTiO_2_ scaffolds after culture for 1, 3, and 5 days.

The adhesion morphologies of MG-63 cells cultivated on the scaffolds were showed (Figure 10). The scaffolds with nTiO_2_ showed a better cell adhesion than that of the scaffolds without nTiO_2_ after cultured for one day. After 3 days of incubation, cells on the scaffolds with nTiO_2_ presented affluent filopodias and were spread extensively on the scaffolds. While the scaffolds without nTiO_2_ had few filopodias. When cultured for 5 days, the cells secreted extracellular matrix (ECM) and formed confluent cells layer on the both of scaffolds. Besides, almost entire region of the scaffolds with nTiO_2_ were covered with the cells and formed a thicker cell layer than that on the scaffolds without nTiO_2_.

**Figure 11:**
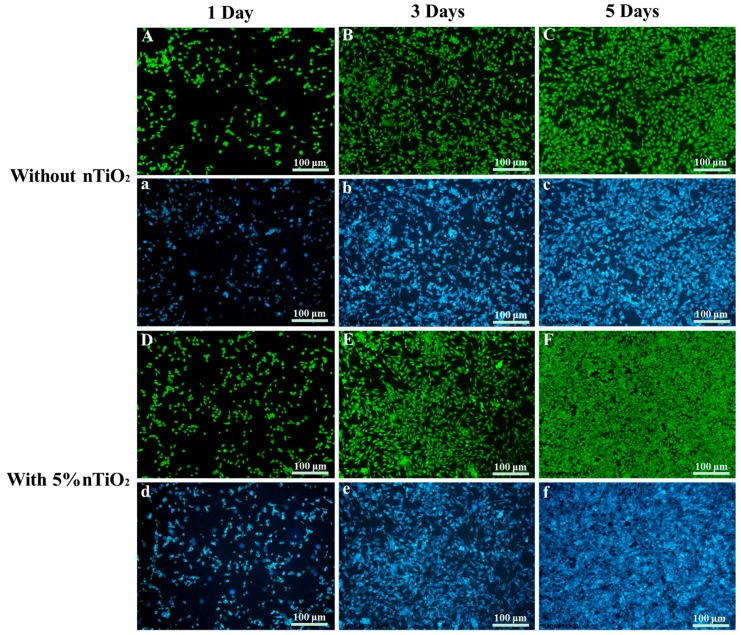
Fluorescence microscopy images (live cells appeared as bright green dots and related cytoblast as purplish blue dots) of MG-63 cells on (**A**–**C** and **a**–**c**) PEEK/PGA and (**D**–**F** and **d**–**f**) PEEK/PGA-5%nTiO_2_ scaffolds after culture for 1, 3, and 5 days.

In order to further illustrate the attachment and proliferation of the MG-63 cells, the cell fluorescence experiment was carried out (Figure 11). The cells on the scaffolds exhibited light green and related cytoblast presented purplish blue. It was apparent that cell number increased with the increase of incubation time. More importantly, the change trend of the cells cultured on the scaffolds with nTiO_2_ was more obvious than the cells cultured on the scaffolds without nTiO_2_. The results indicated that nTiO_2_ was conducive to cellular attachment and spread.

**Figure 12:**
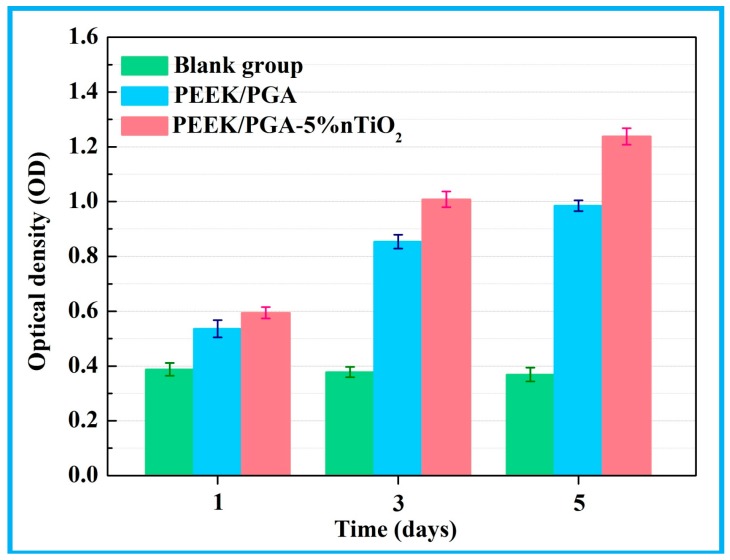
Proliferation of MG-63 cells on PEEK/PGA-5%nTiO_2_ and PEEK/PGA scaffolds after culture for 1, 3, and 5 days.

The cell proliferation on the PEEK/PGA-5%nTiO_2_ was evaluated using MTT assay (Figure 12). Compared with the blank group, it was obvious that both of the scaffolds demonstrated high absorbance values during the whole culture time, which indicated an enhanced cell proliferation. Moreover, it should be noted that the cell density on the scaffolds with nTiO_2_ was higher than that on the scaffolds without nTiO_2_. The results implied that nTiO_2_ could promote the proliferation of MG63 cells. It could be concluded that nTiO_2_ was in favour of cellular proliferation and attachment, which might be due to the favourable biocompatility of nTiO_2_ [47]. Further, the scaffolds with nTiO_2_ were more hydrophilic, which could promote the interaction between cells and scaffolds [48,49].

## 4. Conclusions

PEEK/PGA-nTiO_2_ scaffolds were fabricated via SLS. The polymeric scaffolds with nTiO_2_ possessed significant antibacterial capability. Moreover, their tensile strength and modulus were improved by 35% and 48% with 5 wt % nTiO_2_, respectively. And the hydrophily and thermal stability of the scaffolds were also elevated. Furthermore, MG-63 cell experiments testified that nTiO_2_ could promote cellular attachment and proliferation. Hence, PEEK/PGA scaffolds with nTiO_2_ might be promising implants for tissue engineering.
